# The Use of Massage Therapy for Reducing Pain, Anxiety, and Depression in Oncological Palliative Care Patients: A Narrative Review of the Literature

**DOI:** 10.5402/2011/929868

**Published:** 2011-08-23

**Authors:** Maria Falkensteiner, Franco Mantovan, Irene Müller, Christa Them

**Affiliations:** ^1^Division of Cardiology, Hospital of Bolzano, District Health Bolzano-Südtirol, L. Bühlerstr. 5, 39100 Bolzano, Italy; ^2^Nursing Department, University of Applied Sciences-Verona, Claudiana-Spitalstr. 11, 39031 Brunico, Italy; ^3^Department of Psychiatry, University of Innsbruck, Innsbruck, Austria; ^4^Department of Nursing Sciences and Gerontology, UMIT University, Eduard Wallnöfer-Zentrum1, 6060 Hall in Tirol, Austria

## Abstract

A considerable number of cancer patients use complementary medicine therapies in order to alleviate different symptoms such as pain, anxiety, and depression, occurring in connection with cancer. This paper explores the question to what extent massage therapies are able to reduce the amount of pain, anxiety, and depression. For this purpose, a systematic literature analysis was carried out in the electronic databases and specialist journals. There is already evidence that massage therapies can influence the symptoms of pain, anxiety, and depression in a positive way.

## 1. Introduction

Cancer is the second most frequent cause of death after cardiovascular diseases, leading to unbearable and hardly controllable symptoms in 70 to 80% of the cases, especially when the healing phase is over and the therapy has to be continued with palliative intent [1].

Depending on the type of cancer and the stage of the disease, different physical and mental symptoms can be noted, with pain, anxiety, and depression playing a central role in the care and treatment of critically and terminally ill patients as the disease progresses [2].

In order to alleviate severe pain, opioid analgesics are administered because of their high efficacy and the absence of specific organ toxicity. Despite their good effectiveness, several side effects such as nausea and vomiting, drowsiness, confusion, respiratory depression, antitussive effect, urinary retention, itchiness, constipation, hyperalgesia, tolerance, and dependency can be noted [2, 3].

Due to the progression of cancer and the simultaneous presence of severe pain, there is an increased risk of mental complications such as anxiety and depression [2, 4]. Ten to 20% of oncological patients suffer from a depression [2]. Anxiety disorders can be found with a prevalence of 13 to 79% in this group of patients [5]. Anxiety is a multifactorial disorder which can be connected with other symptoms such as depression, and its pharmacological treatment mainly consists of benzodiazepines, antidepressants, neuroleptics, and opioids [1, 6, 7]. The side effects of these medications include ataxia, respiratory problems, amnesia, cardiac muscle fatigue, psychomotor and cognitive impairments, sleep disorders, and paradoxical reactions [1, 5].

As pharmacological treatment causes a number of side effects, oncological patients frequently turn to complementary medicine therapies as an adjunctive treatment to ease the symptoms [8]. Massage therapies are among the most frequently used complementary treatments. Their effectiveness with regard to the reduction of symptoms of pain, anxiety, and depression in oncological patients has been examined in several studies [8–12]. 

As far as nursing practice is concerned, it is currently difficult to consult scientific works clearly illustrating the influence and effectiveness of this method in oncological patients [8, 13, 14].

## 2. Objective and Questions of the Literature Review

The aim of this literature review is to examine the effectiveness of massage therapy for reducing pain, anxiety, and depression in patients receiving palliative oncological care. 

Questions.

- Can massage therapy reduce the level of pain in patients receiving palliative oncological care?
- Can massage therapy reduce the level of anxiety in patients receiving palliative oncological care?
- Can massage therapy reduce the level of depression in patients receiving palliative oncological care?

## 3. Methodical Approach

In order to respond to the above questions in narrative literature review was conducted from March to July 2010 in the following databases: The Cochrane Library, CINAHL (Cumulative Index to Nursing and Allied Health Literature), PsychInfo (Ebsco), Medline (National Library of Medicine), Embase, Amed (Ebsco), and Tripdatabase. The review was limited to studies published between 2000 and 2010. In addition, the bibliographical references of the authors and the selected journals were examined for further references. The literature was selected according to the inclusion and exclusion criteria stated in Table 1.

## 4. Selection of the Studies

By applying the described strategy, a total of 69 articles were found, 38 of which were relevant hits. After carefully sorting through the relevant hits, the duplicates were removed. Applying the in- and exclusion criteria, 20 articles were found appropriate. 

**Figure 1:**
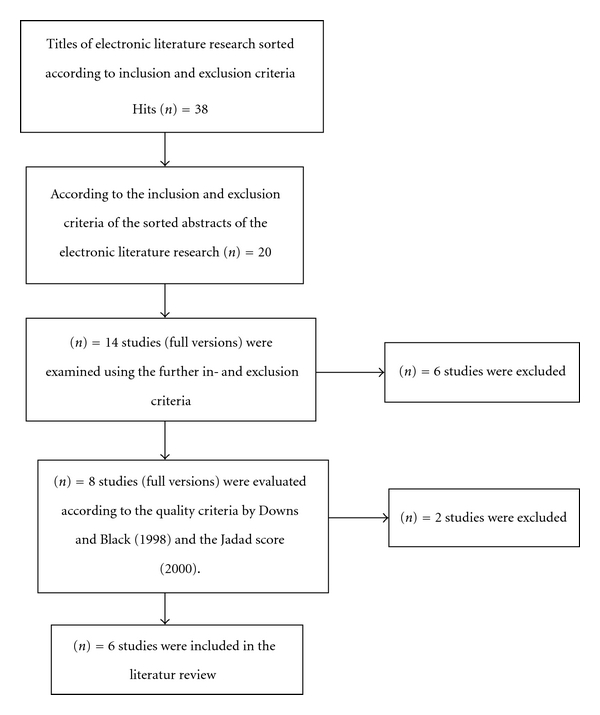
Synthesis of the literature selection (own illustration).

The search strategy and the selection and quality rating of the relevant studies were carried out and analysed separately by two persons in a first step and jointly synthesised and discussed in a second step. The studies that had been found were selected according to the inclusion criteria and the quality criteria of the Jadad Score (2000) for RCTs and the checklist of Downs and Black (1998) for randomised and nonrandomised studies. Using these criteria, six articles were eventually included in the final analysis (see Figure 1).  Table 2 shows the eight Studies excluded after examination of the full versions. The classification of the studies according to evidence level was carried out according to Kunz et al. [15], with four studies corresponding to evidence level I [12, 13, 16, 17] and two to evidence level III [18, 19].

The analysis of the publications and the data extraction was done using a tabular format (see Table 3).

## 5. Results of the Literature Review

### 5.1. Characteristics of the Population

The six selected studies comprised a total of *n* = 1,558 adult oncological patients receiving palliative care.

The six analysed studies included both female and male patients, with female patients accounting for a higher percentage share in four of the examined studies [12, 13, 17, 18]. Male patients outnumbered females in the two other studies [16, 19]. The patients were between 30 and 88 years of age. The average age was between 65 and 66 years in five studies [12, 13, 16, 17, 19]. The average age in the study of Jane et al. [18] was considerably lower, namely, 52 years. The patients suffered from lung, breast, pancreas, prostate, and colorectal cancer [12, 13, 16–19]. All patients were diagnosed with metastases. The studies of Jane et al. [18] and Kutner et al. [13] also included patients diagnosed with bone metastases. 

The probable life expectancy of the patients which took part in the studies was estimated at less than six months. In five studies, the patients received palliative care in a hospice or in an oncological centre [13, 16–19]. Cassileth and Vickers [12] had also included additional patients living and receiving palliative care at home. Kutner et al. [13] found that 49% of the patients were in a relationship.

Four of the selected studies were conducted in the USA [12, 13, 16, 17]. The two other studies were carried out in Asia [18, 19].

### 5.2. Interventions

The patients participating in the different studies mainly received a full-body massage or partial massage [13, 16–18]. Cassileth and Vickers [12] also offered a foot massage or a gentle touch massage. Osaka et al. [19] administered a hand massage only.

### 5.3. Effect of Massage Therapy for Reducing Pain

The symptom of pain was examined in five out of the six included studies [12, 13, 16–18]. The analgesic effect of massage therapy in oncological patients receiving palliative care could be shown in four out of these five studies [12, 13, 16, 18]. In four studies, the amount of pain reduction reached a statistically significant value (see Table 3) [12, 13, 16, 18]. The results also showed that massage therapy yielded a considerably better effect in patients with strong pain perception (VAS >4) [12, 13, 18]. However, Downey et al. [17] could not prove the effectiveness of massage therapy in terminal oncological patients through their study results (see Table 3).

Three out of six studies examined the long-term effects of massage therapy [13, 16, 18]. It is notable that the results turned out to be very divergent. Jane et al. [18] provided the longest followup of all analysed studies with a period of 16 to 18 hours. However, Jane et al. [18] found that massage therapy yielded no statistically significant effect on pain perception after this period (see Table 3). Kutner et al. [13] and Wilkie et al. [16] also analysed the lasting effect of massage therapy, and these authors found the immediate effects to be higher and the longer-term effects to be lower. 

In addition to the lasting effect of massage therapy, Wilkie et al. [16] also examined the change in pain. Wilkie et al. [16] found a transformation from constant pain perception to intermittent or episodic pain perception in 14% of the participating patients. Summing up, it can be stated that massage therapy can achieve a reduction of pain lasting up to 18 hours [13, 16, 18].

Massage therapy shows a favourable effect in both the immediate and the continuous analysis of the results. In order to further support this effect, Kutner et al. [13] and Wilkie et al. [16] studied the patients' consumption of analgesics after they had received massage therapy and compared it with the previous dosage. While the decrease in the consumption of analgesics was not statistically significant, the dosage of analgesics was subject to less fluctuation [16].

### 5.4. The Effect of Massage Therapy for Reducing Anxiety and Depression

The presence of pain can cause anxiety and depressions to develop or to become more pronounced [4]. For this reason, the effect of massage therapy in view of these both disease symptoms was assessed in four out of six studies [12, 13, 18, 19].

The symptom of anxiety was examined in three out of six studies; however, the authors did not give a definition of anxiety [12, 18, 19]. 

The authors found physiological relaxation to be closely connected with the immediate reduction of anxiety, and they also found it to be of importance for a lasting effect [12, 18, 19]. Monitoring the heart and respiratory rate after the respective massage therapy may indicate a relaxation. While Jane et al. [18] were able to note a reduction of the rates, the results were not statistically significant. However, the patients' perception of anxiety immediately after the intervention had decreased statistically significantly (see Table 3) [18].

The study of Osaka et al. [19] was limited to the administration of a hand massage. Despite the short duration of only five minutes, a statistically significant reduction of the perception of anxiety could be achieved (see Table 3). Cassileth and Vickers [12] provided evidence for a considerably higher reduction both with regard to the perception of pain and with feelings of anxiety in those patients who had stated a higher initial value of anxiety (VAS >4) before the intervention.

Physical contact plays an important role in reducing anxiety. During a massage, there is physical contact between the massage therapist (the caregiver) and the patient. A prerequisite for the effectiveness of the intervention is that the patient can accept this close physical contact [16, 17].

### 5.5. The Effect of Massage Therapy for Reducing Depression

The effectiveness of massage therapy for reducing depression and depressive states of mind was analysed in two of the selected studies as a secondary outcome [12, 13]. These two studies provided evidence for an improvement of the depressive mood through massage therapy. However, the authors of these two studies noted that the type of massage and the setting are to be taken into consideration as important influencing factors. The analysis of the results of Cassileth and Vickers [12] showed that a gentle touch massage or full-body massage provides for clearly better results in easing symptoms (*P* = 0.03) than a foot massage. There was no significant deviation (*P* = 0.12) between the results of full-body massage and gentle touch massage. 

Massage therapy thus has a favourable influence with regard to reducing anxiety and depression [13, 18].

None of the six studies found negative effects of massage therapy. There were not any incidents either in patients who already had bone metastases [13, 18]. However, limitations were indicated regarding the administration and the duration of massages. Some patients were unable to find a pleasant position causing the duration of the massage to be shortened [18]. Other patients were in a poor general state of health making it impossible to administer a full-body massage. For this reason, the massage therapist had to be flexible in carrying out the massage and concentrate on a partial massage if necessary [16, 17]. The massage sessions only had to be interrupted, if at all, due to telephone calls or visitors the patient wanted to receive [13].

## 6. Discussion

Massage therapy has proven to reduce the subjectively perceived symptom of pain in oncological patients receiving palliative care. Remission of the symptoms of anxiety and depression, examined secondarily, was also achieved.

Despite the different characteristics of the population, similar results with respect to reducing pain were achieved in four out of six studies [13, 16–18].

The qualitative data gained from the analysed studies has shown that interventions such as massage therapy only seem to be effective if the patient is treated with empathy and if a relationship between the massage therapist and the patient had been formed beforehand [13, 16]. This observation may support the hypothesis that desired or undesired effects of a massage are not only dependent on the interventions themselves but also on the time of the day, the setting, the position of the patient, and the type of massage; in addition, the attitude of the therapist plays an important role [13].

The perception of pain in the analysed studies was found to have different initial values, with the highest initial value being the one in the study of Jane et al. [18]. This study was conducted in Taiwan. While all patients were diagnosed with bone metastases, the cultural aspect may influence the subjective assessment of pain. 

The assumption of Kutner et al. [13] and Wilkie et al. [16] that massage therapy can achieve substantial pain reduction and consequently lower the use of analgesics was not confirmed. Patients require sufficient pharmacological pain treatment; otherwise a state of relaxation before the beginning of the massage treatment cannot be achieved [18].

Only one study [16] found an increase of pain perception as a negative effect. Direct negative effects of massage therapy were not shown in the remaining examined studies. Fellowes et al. [20] note that possible digestive problems might be a negative effect of massage therapy and that patients should therefore be examined for this condition. 

The assumption that massage therapies are to be considered contraindicated with malign tumours because tumour growth and metastasizing may be accelerated was refuted by several authors [8, 16, 18]. In the analysed studies, both the authors and the patients mainly aimed at a full-body massage [12, 13, 16–18]. It had to be noted that some patients were unable to find a comfortable position or that the position needed to be changed permanently, thereby disturbing the massage and reducing its effect [18]. This suggests the conclusion that the duration of a massage plays a crucial role for achieving the desired effect and enabling the patient to experience relaxation during the massage therapy [22]. 

Kutner et al. [22] state that seriously ill patients might associate physical contact and touch with painful invasive techniques such as taking a blood sample. For this reason, the patient should be in a relaxed state before the beginning of the message therapy and thoroughly informed about the massage therapy and the kind of physical contact.

As a matter of principle, thoroughly informing the patients and their relatives is of utmost importance in treating various symptoms of people who are to be provided with palliative care. Apart from information, direct communication gives the patient trust and a feeling of security, thus additionally increasing the amount of self-determination of the patient with respect to the treatment of potential symptoms [8]. A lack of information provided to the patient by the caregivers concerning the effect of massage entails the risk of the patient refusing massage therapy. In addition, the patients might gain the impression that they are robbed of the time they have left in a senseless way [16]. Massage therapy also enables the caregivers and the patients to deepen their relationship through mutual physical contact and to strengthen mutual trust [22]. The importance of thoroughly counselling and informing the patient cannot be estimated high enough.

Offering massage therapy is felt as a relief by hospice and palliative care patients. Patients whose social network is poor especially consider massage therapy a precious offer [25]. Those patients who experience little physical contact, affection and security may be more responsive to massage therapy. Therefore, it should especially be made available for socially isolated patients [9, 26].

Summing up, it can be stated that massage therapy is to be considered a cost-efficient, noninvasive intervention positively influencing and contributing to the reduction of pain, anxiety, and depression in seriously ill cancer patients [12, 17].

## 7. Implications for Research

Further studies are necessary in order to confirm the effectiveness of massage therapy with respect to reducing the symptoms in patients receiving palliative care. Future studies should deal with different kinds of massage therapy in order to be able to provide solid data for nursing practice within the vulnerable group of terminal oncological patients. Furthermore, uniform interventions, assessment instruments, and designs should be used for collecting the results to enable comparability.

## Figures and Tables

**Table 1:** Inclusion and exclusion criteria applied to the literature research (own illustration).

	Inclusion criteria	Exclusion criteria
Population	(i) Oncological patients older than 18 years of age	(i) Oncological patients younger than 18 years of age

	(ii) Advanced disease stage (terminal phase)	(ii) Oncological patients also suffering from a psychosis

Intervention	(i) Massage therapy	(i) Acupuncture and acupressure
	(ii) Full-body massage	(ii) Reflexology
	(iii) Partial massage (iv) Hand massage	(iii) Aroma therapy massage
		(iv) Lymphatic drainage and all other forms of complementary medicine therapies

Outcome	(i) Pain	(i) All other disease symptoms and result parameters
	(ii) Anxiety	
	(iii) Depression	

Setting	(i) Palliative care in hospice facilities, at home or in an oncological centre	(i) Acute and intensive care unit
		(ii) Patients not receiving palliative care

Year of publication	(i) From 2000 to 2010	(i) Before the year 2000

Language	(i) English	(i) All other languages
	(ii) German	
	(iii) Italian	

Key words. Advanced cancer, terminal neoplasms, end-of-life, terminal disease, massage, massage therapy, Swedish massage, hand massage, palliative care, hospice care, end of life care, pain, anxiety, depression, mood.

**Table 2:** Studies excluded after examination of the full versions (own illustration in alphabetical order).

Study	Reason
Ernst [8]	Population comprises oncological children as well as adults. The intervention of massage therapy does not only refer to palliative care but also to curative and rehabilitative care.
Fellowes et al. [20]	This systematic review was published in 2004 and mainly refers to studies published before the year 2000.
Gorman et al. [21]	The aim initially set does not correspond to the results. The results of the initial aim will be published in a future study.
Gray [22]	Literature review of poor methodical quality (interventions and results were only partly stated).
Polubinski and West [23]	Practice report of poor methodical quality (data analysis, presentation of the results and description of the intervention).
Russell et al. [9]	This systematic review includes both children and adult oncological patients.
Smith et al. [24]	The intervention of the massage therapy refers to the characteristics of the massage therapy. The massage therapist is supposed to take into consideration, that is, the type of massage, the position of the patient, and so forth.
Wilkinson et al. [11]	This systematic review refers to adult oncological patients receiving care in any health care facility.

**Table 3:** Table summarising the results of the data extraction (own illustration in alphabetical order).

Author	Design and sample	Intervention	Assessment instrument	Results	Remark
Cassileth, Vickers, [12]	Quasiexperimental study Three groups: (i) classic massage: *n* = 560, (ii) therapeutic touch: *n* = 90, (iii) foot massage: *n* = 585.	Three sessions lasting 30 minutes each with institutionalised patients and lasting 60 minutes with patients living at home.	(i) VAS (for measuring pain, fatigue, stress/anxiety, nausea, and depression)	Immediate effect: (i) VAS** (**reduction of pain): mean average value of change −1.7 (SD ± 2); *P* = 0.05 (ii) VAS (reduction of anxiety**)**: mean average value of change −2.8 (SD ± 2.5) (iii) VAS** (**reduction of depression): mean average value of change −1.2 (SD ± 1.9) VAS >4: highest effect (mean average value of change: pain: −2.9; anxiety: −4; depression: −3)	No randomisation

Downey et al., [17]	Randomised controlled study Three groups: (i) massage: *n* = 56, (ii) meditation: *n* = 56, (iii) control group: *n* = 55.	35-minute massage therapy or meditation	(i) MSAS (Memorial Symptom Assessment Scale): immediate pain reduction	(i) MSAS (immediate pain reduction): *P* = 0.573 Massage therapy does not show a statistically significant reduction of pain.	

Jane et al.; [18]	Observational study: (i) Intervention group: *n* = 30	Administration of a full-body massage. Duration: 45 minutes.	(i) PPI-VAS (present pain intensity using a vertical visual analogue scale: immediate change of pain intensity) (ii) MSF-MPQ (Short-Form McGrill Pain Questionnaire and the Brief Pain Inventory): Quality and localisation of pain (iii) VAS (Anxiety-VAS)	Immediate effect: (i) PPI-VAS: *P* = 0.001 (ii) VAS: *P* = 0.001 Medium-term effect: (i) PPI-VAS and VAS at 15 minutes: *P* < 0.002 (ii) PPI-VAS and VAS at 20 minutes: *P* < 0.000 Long-term effect (at 16–18 hours postintervention**)** (i) MSF-MPQ for pain quality: *P* = 0.08 (ii) MSF-MPQ for pain localisation: *P* = 0.04	Control group missing

Kutner et al., [13]	Multicentred randomised clinical study and meta-analytical trial Two groups: (i) intervention group: *n* = 188, (ii) control group: *n* = 192.	Six full-body massages administered within two weeks. Duration: 30 minutes each.	Immediate effect: (i) MPAC (Memorial Pain Assessment Card 0–10 scale): Immediate change in pain (ii) MPAC (Memorial Pain Assessment Card 0–10 scale): Immediate and lasting effect of mood Long-term effect: (i) BPI (Brief Pain Inventory BPI 0–10 scale): Long-term change in pain	Immediate effect: (i) MPAC (pain): mean average value of change −1.87 (ii) MPAC (mood): mean average value of change −1.58 Long-term effect: (i) BPI (mean average value of pain): mean average value of change −0.33 (ii) BPI (maximum pain intensity): mean average value of change −0.74 (iii) BPI (pain interference): mean average value of change −0.33	

Osaka et al., [19]	Observational study (i) Intervention group: *n* = 34	5-minute massage of the upper extremities	Immediate effect (i) STAI-state-score: Perception of anxiety	Immediate effect (i) STAI-state-score: *P* < 0.001	Control group missing

Wilkie et al., [16]	Randomised controlled pilot study Two groups: (i) intervention group: *n* = 15, (ii) control group: *n* = 14.	Full-body massage twice a week over a period of two weeks. Duration: 45 minutes.	(i) PAT (Pain Assessment Tool, 0–10 Scale)	Immediate effect (i) PAT: *P* < 0.05 (after the first and third massages); *P* < 0.09 (after the fourth massage) Long-term effect (at two weeks postintervention) (i) Perception of pain: transition from constant pain to intermittent episodes of pain in 14% of the patients (ii) Intensity of pain: *P* > 0.26; reduction of pain in 42% of the patients	
